# Risk assessment of novel coronavirus COVID-19 outbreaks in the border areas of southwest China

**DOI:** 10.1097/MD.0000000000029733

**Published:** 2022-07-08

**Authors:** Lihua Chen, Yuanyuan Xiao, Jibo He, Huxing Gao, Jiang Zhao, Shiwen Zhao, Xia Peng

**Affiliations:** a Epidemic Surveillance/Public Health Emergency Response Center, Yunnan Center for Disease Control and Prevention, Kunming, Yunnan, China; b School of Public Health, Kunming Medical University, Kunming, Yunnan, China; c Comprehensive Security Department, Yunnan Provincial Center for Disease Control and Prevention, Kunming, Yunnan, China; d Nutrition and Health Institute, Yunnan Center for Disease Control and Prevention, Kunming, Yunnan, China; e Administrative Office of YNCDC, Kunming, Yunnan, China.

**Keywords:** border areas, hierarchical prevention and control, novel coronavirus, risk assessment

## Abstract

This study aimed to assess the risk of coronavirus disease 2019 in the border areas of southwest China, so as to provide guidance to targeted prevention and control measures in the border areas of different risk levels.

We assessed the dependence of the risk of an outbreak in the southwest China from imported cases on key parameters such as the cumulative number of infectious diseases in the border area of southwest China in the past 3 years; the connectivity of the neighboring countries with China’s Southwest border, including baseline travel numbers, travel frequencies, the effect of travel restrictions, and the length of borders with neighboring countries; the cumulative number of close contacts of coronavirus disease 2019 patients; (iv) the population density in border areas; the efficacy of control measures in border areas; experts estimated risks in border areas based on experience and then given a score; Spearman correlation and Logistic regression models were used to analyze the associated factors of novel coronavirus. According to the correlation of various factors, we assigned values to each parameter, calculated the risk score of each county, and then divided each county into high, medium, and low risk according to the sick score and took different control measure according to different risk levels. Finally, the total risk level was evaluated according to the Harvard disease risk index model.

The number of infectious diseases in the past 3 years, travel numbers, travel frequencies, experts estimated risk score, effect of travel restrictions, and the number of close contacts were associated with the incidence of new coronary pneumonia. It is concluded that bilateral transportation convenience is a risk factor for new coronary pneumonia, (odds ratio = 9.23, 95% confidence interval, 1.99–42.73); the number of observers is a risk factor for new coronary pneumonia (odds ratio = 1.04, 95% confidence interval, 1.00–1.08).

We found that in countries with travel numbers, travel frequencies, and experts' estimated risk scores were the influencing factors of novel coronavirus. The effect of travel restrictions and the cumulative number of close contacts of the case are risk factors for novel coronavirus.

## 1. Introduction

Coronavirus disease 2019 (COVID-19) is a type of pneumonia caused by novel coronavirus (SARS-CoV-2) infection. This disease is highly infectious, and the entire population is generally susceptible.^[1–5]^ In December 2019, SARS-CoV-2-infected pneumonia (COVID-19) first occurred in Wuhan and has rapidly spread out. The novel virus was officially named SARS-CoV-2, with the disease termed as COVID-19.^[6]^ Epidemiological data demonstrated person-to-person transmission in hospital and family settings.^[7,8]^ The high infectivity of COVID-19 resulted in a rapid increase of new cases and a worldwide outbreak.^[9,10]^

On May 9, 2020, the last case was discharged. As of June 30, 11 cases were imported from abroad. Since then, China has implemented surveillance for potential risk factors for the possible COVID-19 epidemic in border areas. Based on the potential impact of the epidemic in the border area, experts conducted risk assessments and developed a hierarchical and zoned prevention and control plan based on different risk levels. Targeted measures had been taken to achieve precise prevention and control. This is to consolidate the effect of early prevention and control and effectively resume work and school.

Border areas play the role of a peripheral barrier and a key defensive strategic position in national security. Therefore, border security is of vital importance to every country. At this stage, China is facing the dual pressures of external import of the new crown pneumonia epidemic and internal defense rebound. Yunnan has 25 border counties bordering Myanmar, Laos, and Vietnam. The border is 4060 km long. Among them, the China–Myanmar border is 1997 km, the China–Laos border is 710 km, and the Sino–Vietnamese border is 1353 km. The high incidence and weak disease prevention and control systems of neighboring countries and special zones, coupled with frequent cross-border movement of people, will result in the continued existence of imported sources of infection from abroad, and the local epidemic of new crown pneumonia has not been effectively controlled, making the risk of secondary infection high.^[3,8,11,12]^ In addition, the virulence of variant strains of foreign new crown pneumonia virus has increased^[11–15]^ and has a tendency to spread further, posing a serious danger to the prevention and control of new crown pneumonia in the border areas of Yunnan Province.

In view of the influencing factors of infectious diseases in border areas, risk assessment is carried out, and according to different risk levels, prevention and control measures are put forward at different levels to provide scientific basis for precise prevention and control of infectious diseases. Scientific assessment of the risk of infectious diseases at the border is very important for effective prevention and control of infectious diseases.^[1–3,16–18]^ In this study, we used the number of cases as a dependent variable, travel frequency, control strength, etc, as independent variables, analyzed the influencing factors of cases, calculated the risk value based on the influencing factors, and assessed the risk of outbreak of imported cases in border areas.^[14,19]^

## 2. Materials and methods

### 2.1. Study design

This study is based on factors that may cause the input and spread of COVID-19 in the border area of Yunnan Province, which includes 8 states and 25 border counties. We used statistical methods to analyze the influencing factors. Then, we carried out risk assessment based on identified influencing factors. Finally, we took hierarchical prevention and control measures based on high-, medium-, and low-risk levels, effectively controlling the spread of epidemics in border areas. So far, there are no new local cases and no spreading epidemics caused by input.

### 2.2. Data collection and management

The case data collected the number of cases between December 22, 2019, and June 30, 2020. The data for neighboring countries were obtained from the official website of the World Health Organization. The number of ports, access roads, and local prevention and control capabilities came from online surveys and local government departments. The incidence was from the “China Disease Prevention and Control Information System,” and the population was from the Bureau of Statistics.

The incidence of infectious diseases is divided into 3 levels (according to strengths and weaknesses); border control ports were divided into 4 levels; floating population, refer to Baibu migration or local monitoring data; population of close contacts; traffic openness is divided into 5 levels; prevention and control capabilities can be divided into 5 levels from weak to strong; border length; population density; disease status; and experts score based on experience.

### 2.3. Statistical analysis

Data collected were input into Epidata 3.1; to ensure the quality of input, we adopted a double-entry strategy. Given the complex sampling design, the survey package in SPSS 18.0 was used for all analyses. A 2-tailed *P* value <.05 was deemed to be significant. The cumulative number of COVID-19 cases as the dependent variable, the cumulative number of infectious diseases in the border area, baseline travel numbers, travel frequencies, the effect of travel restrictions, the length of borders, the cumulative number of close contacts, the population density, the efficacy of control measures, experts estimated risk score, temperature, humidity, wind direction, precipitation, traffic volume, cumulative incidence of neighboring countries, etc, as independent variables. We used Spearman correlation analysis to analyze the factors of COVID-19 in the border area. Logistic regression models were used to identify associated factors of incidence.

1. The risk value is calculated based on the correlation coefficient and the factor incidence and the risk level of each county is determined based on the value.^[20,21]^


$$\begin{array}{l} RI_{riskvalueofeachcounty}=\sum r\times 100\times P,\;\;r\;is\;Pearsoncorrelationcoef\;f\;icient \end{array}$$

*P is* the probability of the occurrence of the factor, P=m/∑m.

2. Comprehensive evaluation of input risk: Harvard disease risk index model is used to evaluate comprehensive input risk.^[22,23]^


$$\begin{array}{l} \begin{array}{ll} & RI=\frac{RS_{1}+RS_{2}+\cdots RS_{n}}{RS_{1}\times P_{1}+RS_{2\;}\times P_{2}+\cdots RS_{n}\times P_{n}}\times 10\\ & RS(score)=R\times w,\;w=r/\sum r,\\ & R=r\times 10,w=r/\sum \beta ,\\ & P=RS/\sum RS, \end{array} \end{array}$$

RI is the daily incidence risk index of new coronary pneumonia, *R* is the impact value, RS_n_ is the impact factor score, and *P*_n_ is the incidence of the impact factor.

3. New coronary pneumonia risk level classification criteria and measures to be taken (Table 1).

**Table 1:** Classification of the risk index of the incidence of COVID-19.

**Index level**	**Danger level**	**Prediction level**
<10	Low risk	Normal, no warning information is issued
10–30	Medium risk	Early warning reminds relevant departments to The early warning reminds relevant departments to pay attention to the COVID-19 epidemic and carry out publicity and education.
>30	High risk	The Center for Disease Control and Prevention conducts COVID-19 epidemic surveillance, prevention and control, and reserves emergency supplies; government departments pay attention to epidemic trends.

COVID-19 = coronavirus disease 2019.

## 3. Results

### 3.1. Spearman correlation

Spearman correlation was used to analyze the correlation display between the incidence of new coronary pneumonia and factors. The number of infectious diseases in the past 3 years, travel numbers, travel frequencies, experts estimated risk score, effect of travel restrictions, and the number of close contacts were associated with the incidence of new coronary pneumonia (*P* < 0.05) (Table 2).

**Table 2:** The relationship between COVID-19 and influencing factors in the border areas of Yunnan Province.

**Index**	**Number of infectious diseases in the past 3 yr**	**Travel numbers**	**Number of close contacts**	**Travel frequencies**	**Control measures control capacity**	**Effect of travel restrictions**	**Experts estimated risk score**
Coefficient β	0.58	0.58	0.79	0.75	0.67	0.17	0.40
*P* value	.002	.002	<.01	<.01	<.01	.02	.04

COVID-19 = coronavirus disease 2019.

### 3.2. Risk levels of input COVID-19 in 25 border counties in Yunnan Province

**Figure 1.**
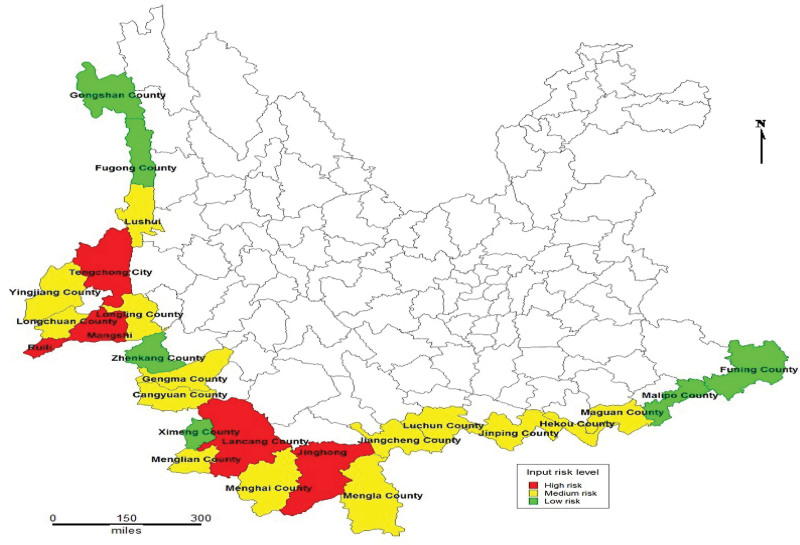
The risk map of input COVID-19 in 25 border counties (cities) in Yunnan. COVID-19 = coronavirus disease 2019.

There are 4 high-risk counties (16%), 10 medium-risk counties (40%), and 11 low-risk counties (44%) in the border area of Yunnan Province (Fig. 1; Table 3).

**Table 3:** Risk levels of input COVID-19 in 25 border counties in Yunnan Province.

**Regional**	**Jinghong**	**Ruili**	**Tengchong**	**Gengma**	**Maguan**	**Longling**	**Menglian**	**Luchun**	**Mengla**
Score	72.04	51.9	44.91	32.39	28.34	27.67	24.37	23.1	21.73
Risk level	High	High	High	High	Medium	Medium	Medium	Medium	Medium
Regional	Lancang	Longchuan	Hekou	Mangshi	Zhenkang	Menghai	Jinping	Yingjiang	Cangyuan
Score	21.11	16.08	15.9	13.42	11.25	9.71	9.44	9.37	9.21
Risk level	Medium	Medium	Medium	Medium	Medium	Low	Low	Low	Low
Regional	Funing	Malipo	Lushui	Fugong	Ximeng	Gongshan	Jiangcheng		
Score	7.73	7.2	5.79	5.45	5.25	4.67	3.97		
Risk level	Low	Low	Low	Low	Low	Low	Low		

COVID-19 = coronavirus disease 2019.

### 3.3. COVID-19 input risk composite value

The risk value was calculated based on the Harvard disease risk model (Table 4).

**Table 4:** COVID-19 input risk composite value.

**Index**	**Score**	**Weight (w**)	**Score**
Number of infectious diseases in the past 3 yr	0.99	0.17	46*
Travel numbers	0.99	0.17	
Number of close contacts	1.74	0.22	
Travel frequencies	1.58	0.21	
Control measures control capacity	1.27	0.19	
Effect of travel restrictions	0.09	0.05	
Experts estimated risk score	0.2	0.05	

* The comprehensive input risk value is 46 scores, and the overall input risk is high risk.

COVID-19 = coronavirus disease 2019.

### 3.4. Logistic regression analysis

**Figure 2.**
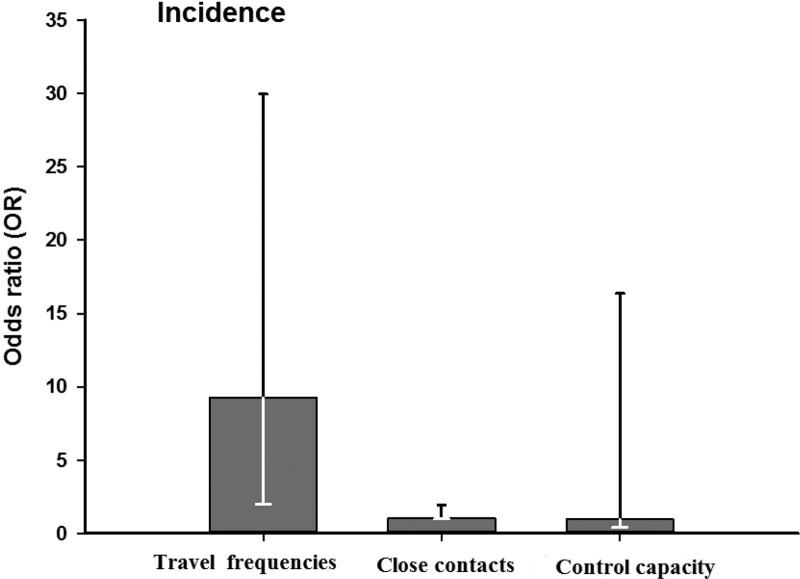
Influencing factors of incidence by binary logistic regression: travel frequencies (<1 in contrast to >4), close contacts (<1 in contrast to >367), and control capacity (<1 in contrast to >7).

It is concluded that bilateral transportation convenience is a risk factor for new coronary pneumonia (odds ratio = 9.23, 95% confidence interval is 1.99–42.73); the number of observers is a risk factor for new coronary pneumonia (odds ratio = 1.04, 95% confidence interval, 1.00–1.08) (Fig. 2).

Figure 2 shows the incidence of infectious diseases in the past (0 in contrast to 2), personnel movement (400 ≤ in contrast to ≥3000), close contacts (0 in contrast to 367), bilateral transportation (0 in contrast to 4), control capacity (0 in contrast to 5), number of ports (1 ≤in contrast to ≥10), and expert score (4 ≤ in contrast to ≥7).

## 4. Discussion

Spearman relevant analysis shows that 7 factors, including the incidence of infectious diseases, the flow of people, the number of tourists, etc, are the influencing factors of COVID-19 in the border areas of China. Further risk assessment based on the influencing factors showed that Ruili, Jinghong, and other places were high-risk areas, Gengma and other areas were medium-risk areas, Menghai and other areas were low-risk areas, and different prevention and control measures were adopted according to different risk levels.

Regression analysis showed that the more open bilateral traffic is, the higher the risk is. Every doubling of traffic openness increases the risk of illness by 9.23 times; the more people who may be exposed to cases and are under observation, the higher the risk of illness is. Every time the number of observers doubles, the risk of illness increases by 1.04 times.

Past epidemic analysis has shown that^[24]^ Ruili and Jinghong have also experienced cross-border imported infectious diseases in the past, with more imported cases and higher frequency.^[25]^ Consistent with the results of this risk assessment, Ruili City, Jinghong City, and other places are high-risk areas. The reasons for the analysis are as follow: Yunnan has always been the area with the most serious imported infectious diseases. Ruili is also the port with the largest throughput of inbound passengers. In 2015, the number of inbound tourists reached 8.38 million. The border trade area of Ruili City is the largest land port and cargo distribution center for Sino–Myanmar trade.^[3,26]^ In March 2021, a COVID-19 epidemic occurred in Ruili, and the first case occurred in the border trade zone.

The prevention and control measures we recommend taking in high-risk areas are as follow: first,implement traffic control on outbound vehicles. Those who really need to leave high-risk areas must undergo a nucleic acid test for COVID-19 within 24 hours and leave after the report is negative. Second, communities where cases of COVID-19 occur need to be blocked.^[27,28]^ All members of the community should undergo nucleic acid testing and vaccination, and vaccination for all members has priority over nucleic acid testing.^[29–32]^ Third, after entering the country, they must be isolated for 14 days, and then at home for 7 days. Isolation period: on the 4th, 7th, 14th, and 21st day, the COVID-19 nucleic acid test will be carried out. If the result is negative, the quarantine can be lifted.^[29,30,33]^ Fourth, tracking, isolation, and nucleic acid testing of close contacts. Fifth, many experts believe that attention should be paid to prevention and control in the southwest border area. In particular, Yunnan has a long border, a lot of land port traffic, and many neighboring countries. Yunnan has always been an area with more imported infectious diseases.^[18,32]^ Jiang Qingwu^[30]^ suggested that vaccination should be given priority in China’s border ports.

There were 15 imported cases by plane. In the early days of the outbreak, all people entering by air were quarantined for 14 days, and the risk of spreading cases from air import was low.

Several limitations of this study should be noted. First, our research was based on cross-sectional nature, thus, causal inference cannot be reached. Second, this study only assesses the risk of border areas in Yunnan Province, and whether it is suitable for other provinces, cities, and regions, in-depth research and evaluation are required. The risk level is changing. When the incidence of infectious diseases in neighboring countries increases or the port opening and other factors change, the port risk level and epidemic prevention measures should be adjusted in time.

### Author contributions

Data curation: Jibo He, Huxing Gao, Jiang Zhao. Funding acquisition: Shiwen Zhao. Writing original draft: Lihua Chen. Writing review and editing: YuanyuanXiao, Xia Peng.
